# Establishment of a mouse intrauterine adhesion model via transvaginal mechanical injury guided by abdominal ultrasound

**DOI:** 10.1371/journal.pone.0347963

**Published:** 2026-06-05

**Authors:** Xuan Liu, Panpan Yuan, Xindi Zhang, Xin Liu, Caixiang Li, Hui Zhao, Yinci Zhang, Fan Li, Ping Zhou

**Affiliations:** 1 Department of Obstetrics and Gynecology, The First Affiliated Hospital of Anhui University of Science and Technology, Huainan, China; 2 Department of Ultrasound, The First Affiliated Hospital of Anhui University of Science and Technology, Huainan, China; 3 Anhui University of Science and Technology, Huainan, China; 4 Academic Research Division, The First Affiliated Hospital of Anhui University of Science and Technology, Huainan, China; PLOS: Public Library of Science, UNITED STATES OF AMERICA

## Abstract

**Objective:**

To establish a clinically relevant mouse model of intrauterine adhesion (IUA) using a minimally invasive approach that combines real-time ultrasound guidance with controlled transvaginal mechanical injury.

**Methods:**

Forty female C57 mice in estrus were randomly assigned to four groups (n = 10 each): ultrasound-guided transvaginal mechanical injury, laparotomy mechanical injury, absolute ethanol, and laparotomy control. Fourteen days after surgery, mice were euthanized and uterine tissue was collected for histological evaluation. Hematoxylin–eosin staining was used to assess endometrial morphology and quantify gland density, while Masson trichrome staining was used to fibrosis. Early and late experimental complications were monitored throughout the study.

**Results:**

The ultrasound-guided transvaginal mechanical injury, laparotomy mechanical injury, and absolute ethanol groups displayed characteristic IUA features, including disrupted endometrial architecture, reduced gland numbers, and significantly increased fibrotic areas compared with controls (P < 0.05). Among the intervention groups, the ultrasound-guided transvaginal group showed the lowest incidence of both early and late complications.

**Conclusion:**

Ultrasound-guided transvaginal mechanical injury successfully established a mouse model of IUA. This approach avoids peritoneal and full-thickness uterine damage, is safe, produces few complications, and partially mimics key aspects of the mechanical injury associated with clinical IUA, providing a reliable platform for future pathogenesis and therapeutic studies.

## 1 Introduction

Intrauterine adhesion (IUA) is a fibrotic lesion that arises when the basal layer of the endometrium is damaged, leading to partial or complete closure of the uterine cavity and endometrial dysfunction. It is primarily caused by iatrogenic trauma from procedures such as induced abortion and postabortion curettage [1–3]. Clinically, IUA often presents with hypomenorrhea, amenorrhea, and reproductive dysfunction [4]. The mainstay of treatment is hysteroscopic adhesiolysis; however, postoperative readhesion is common. Although strategies such as physical barriers have been employed, their preventative effects remain unsatisfactory [5,6]. Therefore, there is a pressing need to develop novel therapeutic approaches.

The pathogenesis of IUA has not been fully elucidated, and most studies have relied on animal models. Thus, establishing accurate and stable animal models of IUA is essential for investigating its pathogenesis and potential therapeutic strategies. Various modeling methods, including mechanical injury, chemical injury, and combinations of mechanical injury and infection, have been proposed [7–11]. These approaches typically involve laparotomy and uterine incision, which not only damage the peritoneum but also injure the uterine serosal and muscle layers, producing pathological changes that differ significantly from common clinical etiologies [2,7–12].

Some researchers have adopted minimally invasive techniques, accessing the uterine cavity via the cervix, which resembles traditional curettage procedures. However, this approach depends heavily on the operator’s experience and tactile feedback, making it technically challenging [13,14]. The degree and location of endometrial damage are difficult to control precisely, increasing the risk of uterine perforation, modeling failure, and animal mortality.

With advancements in ultrasound technology, its application in obstetrics and gynecology has become widespread. Compared with traditional dilation and curettage, ultrasound-guided procedures provide shorter operation times, higher single-procedure success rates, and fewer complications, making it a common clinical practice [15,16]. Additionally, its non-invasive and visual nature has prompted researchers to adopt ultrasound guidance in animal studies to improve experimental safety and accuracy [17].

To better assess IUA development, the purpose of the present study was to establish a clinically relevant mouse model of intrauterine adhesion using a minimally invasive approach that combines real-time ultrasound guidance with controlled mechanical endometrial injury.

## 2 Materials & methods

### 2.1 Materials

#### 2.1.1 Animals.

A total of 40 specific pathogen-free (SPF) female C57 mice (8 weeks old, approximately 20 g each) were obtained from Hangzhou Ziyuan Experimental Animal Science and Technology Co., Ltd. (production license: SCXK [Zhe] 2019−0004; use license: SYXK [Wan] 2023−001). The SPF animal facility was maintained at 24–26 °C with a relative humidity of 57–63%. Animals had ad libitum access to food and water.

#### 2.1.2 Ethical statement.

All experimental procedures, including euthanasia, were performed in full compliance with ethical guidelines for animal research and approved by the Ethics Committee of the First Affiliated Hospital, Anhui University of Science and Technology (approval No. 2022-KY-013–001).

#### 2.1.3 Reagents.

The reagents used in this study included the H&E HD staining kit (Servicebio), Masson staining kit (Servicebio), ethanol (SCRC), xylene (SCRC), and normal butanol (SCRC).

### 2.2 Methods

#### 2.2.1 Establishment of a mouse IUA model.

Vaginal smears were performed to determine the estrous cycle stages of the mice. Forty nonpregnant female C57 mice in estrus were then selected and randomly assigned to four groups using a random number table: the ultrasound-guided transvaginal mechanical injury group (n = 10), the laparotomy mechanical injury group (n = 10), the absolute ethanol group (n = 10), and the laparotomy control group (n = 10).

Anesthesia was induced with isoflurane, and effective anesthesia was confirmed by observing a decrease in respiratory rate, complete muscle relaxation, loss of the eyelid reflex, and absence of response to painful stimuli. The abdominal skin was prepared, and the mice were secured on an operating table equipped with a constant-temperature heating pad. Surgical procedures, specific to each modeling method, were then performed under strict aseptic conditions.

**2.2.1.1 Ultrasound-guided transvaginal mechanical injury group:** An RS80A Samsung Medison two-dimensional ultrasound system with a 5–12 MHz transducer was used. After applying ultrasound gel to the abdomen, the transducer was positioned to determine the location and dimensions of the uterus. In nonpregnant mice, the average uterine horn diameter was approximately (1.5–2.0 mm), with an endometrial thickness of (0.3–0.5 mm) as determined by preoperative ultrasound.

**Fig 1:**
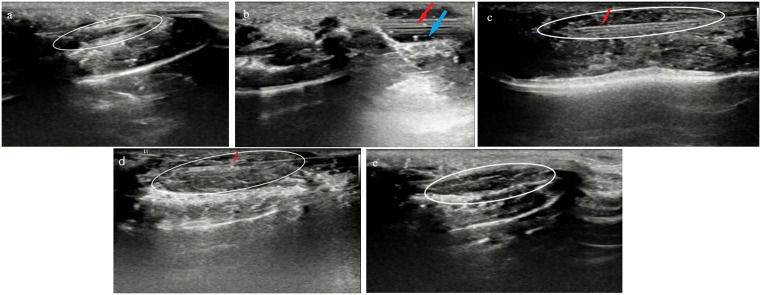
Ultrasound-guided transvaginal mechanical injury procedure in mice. (a) Uterine cavity before injury; (b) Curette entering the vagina; (c) Curette entering the left uterine cavity; (d) Curette entering the right uterine cavity; (e) Uterine cavity after mechanical injury. The white frame indicates the uterine cavity. The red arrow points to the curette. The blue arrow indicates points to vagina.

A small curette (outer diameter: (0.8 mm)) was gently advanced through the vagina to the cervix. Under ultrasound guidance, the left uterine cavity was accessed, and the depth (8–10 mm) was determined. Endometrial scratching was then performed by gently rotating and retracting the curette along the endometrial surface for (3–5 passes), producing an estimated injury length of (6–8 mm). The same procedure was subsequently applied to the contralateral uterine horn. Throughout the procedure, the curette tip and uterine wall were continuously visualized to ensure uniformity of injury and avoid perforation. After the procedure, the vaginal introitus was gently swabbed with povidone-iodine to remove any residual endometrial tissue and sterilize the area. (Fig 1).

**2.2.1.2 Laparotomy mechanical injury group:** The mechanical injury method described by Xi et al. was referenced and modified [11]. The abdomen was sterilized with iodophor followed by 75% alcohol. A 2–3 cm longitudinal incision was made along the midline of the skin, 3–4 cm above the vaginal opening, to expose the underlying muscle. A 2–3 cm longitudinal incision was then made along the linea alba to access the abdominal cavity. The left fat layer was carefully dissected to locate the uterus.

A small incision was made in the uterus approximately 0.5 cm below the ovary, and a small curette was inserted to perform curettage until the uterus appeared translucent. During the procedure, the surrounding fat was kept moist with normal saline. After curettage, the abdominal cavity was flushed with diluted iodophor. The right uterine horn was subjected to the same procedure. Finally, both uterine horns were returned to their normal anatomical positions, and the incisions were closed in layers with absorbable sutures.

**2.2.1.3 Absolute ethanol group:** The absolute ethanol method described by Qi et al. was adopted and modified [18]. Following the surgical steps described above, the left uterus was located, and two vascular clamps were applied to the proximal and distal ends to create a closed uterine cavity. Absolute ethanol was then injected into the cavity until full, maintained for 3 minutes, and subsequently withdrawn. The uterine cavity was flushed with normal saline, and the vascular clamps were removed. The right uterine horn underwent the same procedure. Finally, both uterine horns were returned to their normal positions, and the incisions were closed in layers using absorbable sutures.

**2.2.1.4 The laparotomy control group:** The abdominal cavity of each mouse was exposed using the same surgical approach, and the bilateral uterine horns were exteriorized without any intervention. Then, the uterus was returned, and the abdominal layers were closed in the same way described above. The mice were euthanized 14 days after modeling, and endometrial tissue was collected from the right uterus for subsequent analysis. Euthanasia was performed under deep isoflurane anesthesia, followed by cardiac perfusion fixation, in accordance with internationally accepted guidelines for the humane sacrifice of laboratory animals.

**Fig 2:**
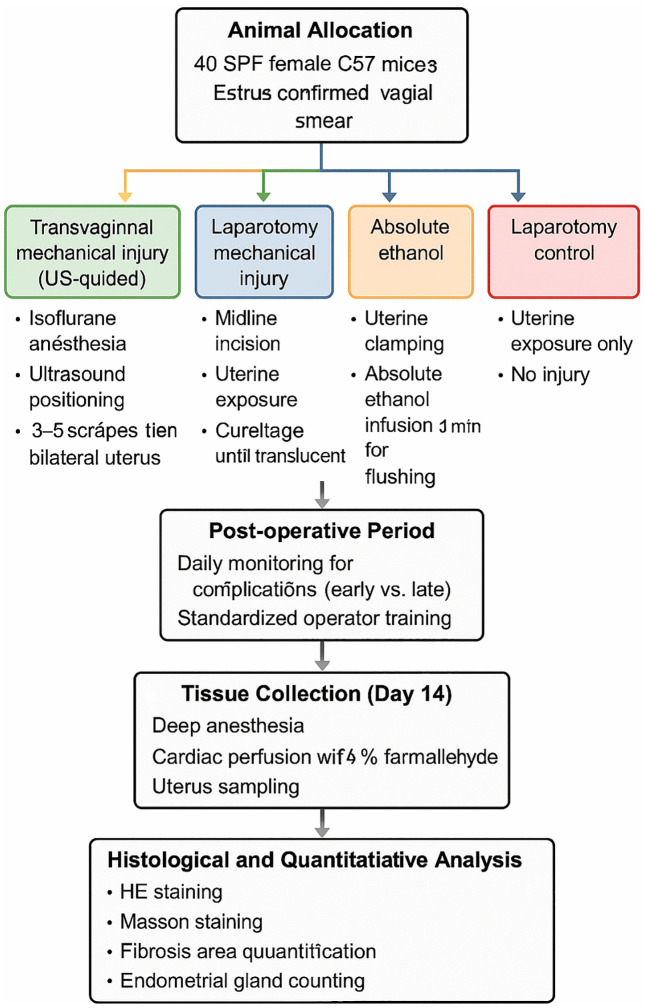
Schematic overview of the experimental design and workflow of the mouse IUA model.

Fig 2 illustrates the overall experimental workflow used to establish and evaluate a mouse intrauterine adhesion (IUA) model. Forty SPF female C57 mice in estrus were randomly assigned to four groups: ultrasound-guided transvaginal mechanical injury, laparotomy mechanical injury, absolute ethanol injury, and laparotomy control. After standardized modeling procedures, all animals underwent postoperative monitoring followed by tissue collection on Day 14 via deep anesthesia and cardiac perfusion fixation. Subsequent histological and quantitative analyses included HE staining, Masson staining, fibrosis assessment, and endometrial gland counting.

#### 2.2.2 Tissue collection and fixation.

Mice were deeply anesthetized using isoflurane until complete loss of reflexes was confirmed, including absence of response to painful stimuli. Euthanasia was then completed by transcardial perfusion, ensuring rapid and humane death prior to tissue collection. After deeply anesthetizing mice, the mouse uterine tissues were obtained via rapid sampling following cardiac perfusion [19]. Although direct immersion of tissues in fixative solutions allows adequate fixation, the fixative does not reach all areas of tissues at the same rate. Moreover, tissue hypoxia often occurs before the fixative enters. Therefore, the use of the circulatory system to directly perfuse the fixative allows the chemical to rapidly reach all parts of the organism through the natural vascular network to achieve whole-body fixation. In this study, 4% paraformaldehyde was perfused through the vascular system for optimal fixation of the mouse uterus.

#### 2.2.3 Operator training and standardization.

All operators completed structured training, including ultrasound identification of uterine horns and supervised practice of transvaginal insertion. Proficiency was confirmed before animal procedures, and standardized insertion depth and ultrasound positioning were documented to ensure reproducibility.

### 2.3 Histological analysis

#### 2.3.1 Hematoxylin and eosin (HE) staining.

To assess histopathological alterations in the murine endometrium 14 days’ post-surgery, paraffin-embedded uterine sections were processed as follows. Sections were dewaxed in laboratory’s standard dewaxing solutions I and II for 20 min each at room temperature, followed by immersion in absolute ethanol I, absolute ethanol II, and 75% ethanol for 5 min each, then rinsed with running tap water. After a 1 min pretreatment, sections were stained with hematoxylin for 3–5 min, rinsed with tap water, differentiated, and blued. Sections were then placed in 95% ethanol for 1 min and stained with eosin for 15 s. Sequential dehydration was performed in absolute ethanol I–III, normal butanol I–II, and xylene I–II for 2 min each. Sections were mounted with neutral gum.Images were captured at 40× and 200 × magnification. Histopathological features—including congestion, stasis, hemorrhage, edema, degeneration, necrosis, hyperplasia, fibrosis, granulation tissue formation, and inflammatory cell infiltration—were assessed under light microscope. For quantification, three random 200 × fields per section were selected, and the number of endometrial glands per field was counted to obtain an average value.

#### 2.3.2 Masson staining.

Sections were subsequently transferred to Masson F for 20–30 s, differentiated briefly in 1% glacial acetic acid, and dehydrated in two changes of absolute ethanol, followed by 100% ethanol and xylene for 5 min each. Sections were mounted with neutral resin.

To evaluate endometrial fibrosis 14 days’ post-surgery, paraffin sections were sequentially dewaxed in standard dewaxing solutions I and II for 20 min each at room temperature, followed by immersion in absolute ethanol I, absolute ethanol II, and 75% ethanol for 5 min each, and rinsed, with tap water and stained with a 1:1 mixture of Masson B and Masson C for 1 min. Sections were then immersed in Masson A solution overnight, rinsed with tap water, and stained with a 1:1 mixture of Masson B and Masson C for 1 min. After rinsing, differentiation was performed for several seconds, followed by immersion in Masson D for 6 min, rinsing, and treatment with Masson E for 1 min. Sections were subsequently transferred to Masson F for 20–30 s, differentiated briefly in 1% glacial acetic acid, and dehydrated in two changes of absolute ethanol, followed by 100% ethanol and xylene for 5 min each. Sections were mounted with neutral gum. Three random 200 × fields per section were analyzed, and the percentage of fibrotic area was calculated and averaged.

#### 2.3.3 Quantification of endometrial glands.

Tissue sections were scanned using a PANNORAMIC panoramic slide scanner, and endometrial glandular regions were imaged at 200 × magnification. Image-Pro Plus 6.0 software was used for analysis, with measurements standardized to millimeters. Within three 200 × fields per slide, the number of endometrial glands and tissue area were quantified. The number of glands per unit area was calculated as the ratio of gland count to tissue area.

### 2.4 Assessment of experimental complications

To systematically evaluate procedure-related complications, all mice were closely monitored from the time of surgery until sample collection. Complications were categorized as early (occurring intraoperatively or within 24 hours) or late (occurring after 24 hours and before sacrifice). Early complications included uterine perforation, ethanol extravasation, significant hemorrhage, spasms, convulsions, and procedure-related mortality. Late complications included wound infection, uterine effusion, intestinal necrosis, and adhesions of the uterus to surrounding tissues.

Assessment was conducted through real-time intraoperative observation, twice-daily postoperative monitoring, and macroscopic examination at necropsy. All complications were recorded, and their incidence was compared among groups. When procedure-related mortality occurred, replacement animals were added to maintain group sizes.

### 2.5 Statistical analysis

Data analysis was performed using SPSS version 25.0. Measurement variables were presented as mean ± standard deviation ($\bar{x}$ ± s) after confirming normal distribution. One-way analysis of variance (ANOVA) was applied to compare differences among groups following the homogeneity of variance test, and post hoc pairwise comparisons were conducted using the least significant difference (LSD) method. A P-value of less than 0.05 was considered statistically significant. Categorical variables were expressed as percentages (%) and evaluated using Fisher’s exact test.

## 3 Results

### 3.1 Evaluation of IUA models

#### 3.1.1 Histopathological changes in the uterus of the IUA mouse model after HE staining.

**Ultrasound-guided transvaginal mechanical injury group**: Uterine tissue exhibited extensive shedding of endometrial epithelial cells (yellow arrows). Several endometrial and glandular epithelial cells showed hydropic degeneration (red arrows), characterized by cellular swelling and lightly stained, loose cytoplasm. The endometrial lamina propria displayed a scarcity of uterine glands, accompanied by edema (purple arrows), loosely arranged connective tissue and stromal cells, and a mild degree of granulocytic punctate infiltration (blue arrows). Some uterine glands underwent mild dilation (dark blue arrows), with sparse eosinophilic material in the glandular lumen (gray arrows). The myometrium showed an irregular arrangement of smooth muscle, with no other significant abnormalities observed (Fig 2A).

**Laparotomy mechanical injury group:** Uterine tissue showed minor shedding of endometrial epithelial cells (yellow arrows) and hydropic degeneration in a few glandular epithelial cells (red arrows), presenting as cellular swelling with lightly stained, loose cytoplasm. Punctate necrosis with karyopyknosis was observed in some endometrial and glandular epithelial cells (black arrows). The endometrial lamina propria contained a few uterine glands with edema (purple arrows), and connective tissue and stromal cells were loosely arranged, accompanied by scattered lymphocytes and granulocyte infiltration (blue arrows). Uterine glands exhibited mild dilation (dark blue arrows). The myometrium displayed an orderly arrangement of smooth muscle, with no other notable abnormalities (Fig 2B).

**Absolute ethanol group:** Uterine tissue and cavity showed minor shedding of endometrial epithelial cells (light green arrows) and hydropic degeneration of some glandular epithelial cells (red arrows), with cellular swelling, light staining, and loose cytoplasm. Punctate necrosis with pyknotic and fragmented nuclei was observed in many endometrial and glandular epithelial cells (black arrows). The lamina propria contained abundant stromal cells and blood vessels, with mild granulocyte infiltration (blue arrows). Occasional scant eosinophilic material was observed in the glandular lumen (gray arrows). The myometrium exhibited an orderly arrangement of smooth muscle, with no other significant abnormalities (Fig 2C).

**Fig 3:**
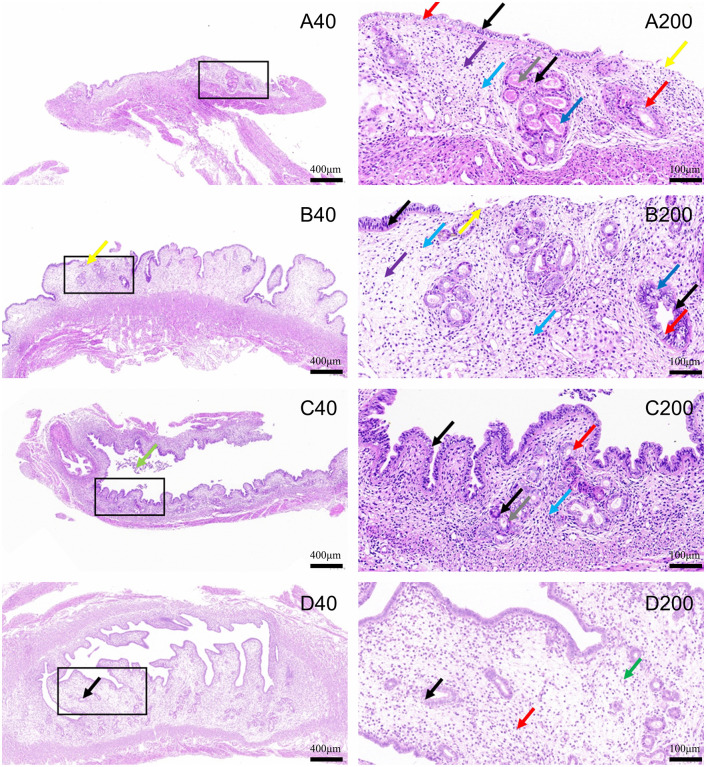
Histopathological analysis of HE-stained uterine sections following different modeling methods. **(A)** Ultrasound-guided transvaginal mechanical injury group; **(B)** Laparotomy mechanical injury group; **(C)** Absolute ethanol group; **(D)** Laparotomy control group. Numbers indicate microscopic magnification, and the black box highlights the area shown at higher magnification.

**Laparotomy control group:** The endometrial epithelium maintained structural integrity, with the presence of tubular uterine glands. These glands were abundant, sparsely arranged, and unevenly distributed. A few endometrial epithelial cells and many glandular epithelial cells showed pale staining and loose cytoplasm (black arrows). The lamina propria was well defined, with loosely arranged connective tissue and occasional granulocyte infiltration (red arrows). The myometrium displayed vascular dilation (green arrows) (Fig 3D).

#### 3.1.2 Histopathological changes in the uterus of the mouse IUA model after Masson staining.

**Fig 4:**
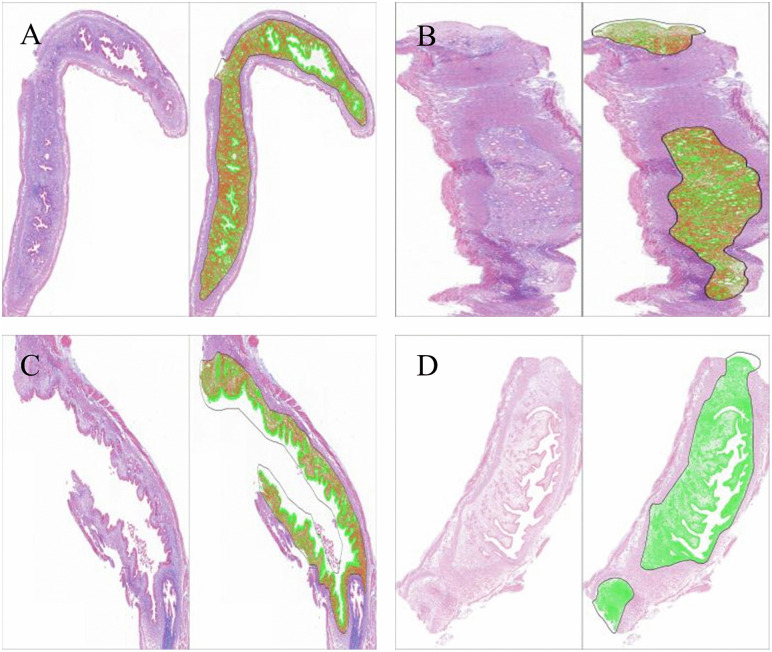
Masson staining of uterine tissue following different modeling methods. (A) Ultrasound-guided transvaginal mechanical injury group; (B) Laparotomy mechanical injury group; (C) Absolute ethanol group; (D) Laparotomy control group.

In the laparotomy control group, the endometrial and myometrial layers showed intact structures and regular morphology, with an orderly arrangement of collagen fibers in the endometrium and stroma. In groups A, B, and C, the endometrium was thin or even absent, and abundant collagen fibers were found in the endometrium and stroma (Fig 4).

#### 3.1.3 Comparison of different endometrial indicators.

**Fig 5:**
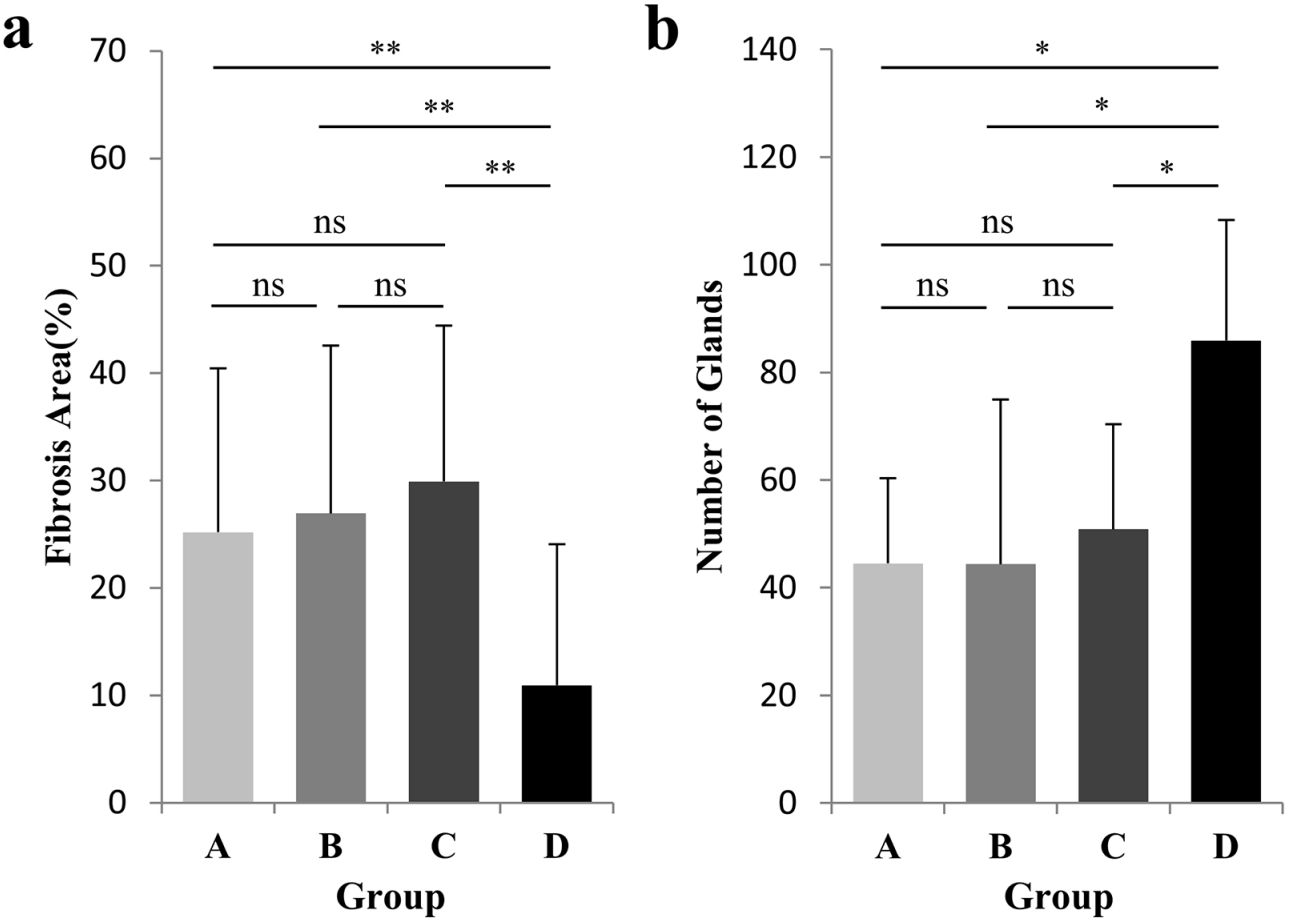
Comparison of endometrial indicators among groups. (a) Percentage of endometrial fibrosis area; (b) Number of endometrial glands (n = 10, mean ± SD; ns, not significant; *P < 0.05, **P < 0.01). Groups: A, ultrasound-guided transvaginal mechanical injury; B, laparotomy mechanical injury; C, absolute ethanol; D, laparotomy control.

The percentage of the endometrial fibrosis area was significantly higher in groups A, B, and C compared to group D, while the number of uterine glands was significantly lower in these groups (P < 0.05). No significant differences were observed in any of the indicators among groups A, B, and C (P > 0.05) (Fig 5).

### 3.2 Experimental complications

The complications observed in this study were categorized as early or late.

#### 3.2.1 Early complications.

Early complications included uterine perforation, ethanol extravasation, and significant hemorrhage resulting from procedural difficulties. Ethanol extravasation could induce spasms, convulsions, and even mortality in mice, while peritoneal damage during laparotomy led to hemorrhage and subsequent mortality in some cases. Specifically, one mouse in Group B and two mice in Group C died. To maintain group sizes, three additional mice were subjected to the same procedures, strictly following the modeling methods of their respective groups.

The incidence of early complications in Group A was significantly lower than in Group C (P < 0.05). Although the total number of early complications in Group A was lower than in Group B, the difference was not statistically significant, likely due to the limited sample size (Table 1).

**Table 1:** Comparison of early complications.

Group	n	Total number of early complications	Uterine perforation	Spasms/ Convulsions	Hemorrhage (0.005–0.02 mL)	Death
A	10	0	0	0	0	0
B	11	4	1	0	2	1
C	12	5	0	2	1	2
D	10	0	0	0	0	0
*P value*		0.01				

A: Transvaginal mechanical injury guided by abdominal ultrasound group. B: Laparotomy mechanical injury group. C: Absolute ethanol group. D: Laparotomy control group.

#### 3.2.2 Late complications.

Late complications, including wound infection, uterine effusion, intestinal necrosis, and adhesions of the uterus to surrounding tissues, occurred between the time of surgery and sample collection. Infections and uterine adhesions were strongly associated with open abdominal surgery. The number of late complications in Group A was lower than in Groups B and C; however, this difference was not statistically significant, likely due to the limited sample size (P > 0.05) (Table 2).

**Table 2:** Comparison of late complications.

Group	n	Total number of late complications	Infection	Uterine effusion	Intestinal necrosis	Adhesion of the uterus to surrounding tissues
A	10	1	0	1	0	0
B	10	6	2	2	1	1
C	10	5	0	3	0	2
D	10	2	1	0	1	0
*P value*		0.08				

A: Transvaginal mechanical injury group guided by abdominal ultrasound. B: Laparotomy mechanical injury group. C: Absolute ethanol group. D: Laparotomy control group.

## 4 Discussion

The endometrium, comprising the functionalis and basalis layers, exhibits robust regenerative capacity. The functionalis layer undergoes cyclical changes under the influence of sex hormones, whereas the basalis layer drives endometrial regeneration. After most physiological injuries, the endometrium can repair itself. However, damage to the basalis layer or deeper tissues, such as that occurring after postpartum or post-abortion curettage, can lead to non-regenerative healing. In such cases, the stroma is replaced by fibrous connective tissue, resulting in adhesion of opposing endometrial surfaces, reduced uterine cavity volume, and endometrial dysfunction. These changes decrease fertility and cause menstrual abnormalities [3,19,20]. Current treatments for intrauterine adhesions (IUA) often fail to achieve ideal outcomes [21,22]. In addition, recent findings have highlighted the potential regulatory role of ghrelin in modulating inflammation and fibrosis during IUA development, offering new insights into the biological mechanisms underlying adhesion formation [23].

To clarify IUA pathogenesis and address therapeutic limitations, various animal models have been developed. The absolute ethanol perfusion method provides consistent and stable model establishment; however, chemical injury produces corrosive pathological changes that do not reflect the progression of mechanically induced IUA, limiting its relevance for pathogenesis studies [9]. Conventional mechanical injury models typically involve laparotomy to expose the abdominal cavity, followed by uterine incision, which damages the myometrium and serosa [8,11,19,20]. This extent of injury differs from common clinical etiologies, making these models less representative of IUA pathology. Furthermore, laparotomy is technically challenging and increases the risk of confounding factors.

Previous studies [13] established IUA models in mice via transcervical mechanical injury combined with infection following cervical relaxation. However, without visualization, the precise injury site was difficult to determine, and the extent of endometrial scraping was challenging to control. This often led to additional damage, such as uterine perforation, resulting in inaccurate model outcomes and unnecessary animal use. Clinically, traditional curettage can severely damage the uterine wall and cervix or be incomplete.

With advances in ultrasound technology, guided procedures have improved safety and efficacy [16]. Ultrasound is non-invasive, allows visualization and repeated examination, and is increasingly applied in animal studies [17,23]. Nevertheless, reports of ultrasound-guided evaluation in IUA animal models remain limited. In this study, we used transvaginal mechanical injury guided by abdominal ultrasound to establish an IUA model and compared it with laparotomy mechanical injury, absolute ethanol, and laparotomy control groups to evaluate modeling efficacy and associated advantages and disadvantages.

Currently, there is no unified standard for evaluating IUA models. Most studies use endometrial thickness, gland count, and degree of fibrosis as indicators [22]. Mild superficial endometrial injury can be repaired within seven days, making models unstable. Severe basal layer damage, however, hinders regeneration and results in fibrosis, reduced uterine cavity volume, and IUA formation within approximately 14 days [8–10,24].

Our results demonstrated that the ultrasound-guided transvaginal mechanical injury group exhibited endometrial thinning, pronounced inflammation, a marked reduction in gland number, and severe fibrosis, consistent with IUA characteristics. No significant differences in gland counts or fibrosis areas were observed among the three IUA models, indicating similar modeling efficacy. Notably, the ultrasound-guided group presented the fewest experimental complications. In contrast, the absolute ethanol group was prone to uterine effusion, adhesions, and ethanol extravasation, causing convulsions, spasms, or mortality. Avoiding laparotomy in the ultrasound-guided group reduced the risk of early complications, such as procedure-induced hemorrhage, and late complications, including wound infection and adhesions.

This method preserves full-thickness uterine integrity, producing pathological changes that closely resemble clinical scenarios. Ultrasound guidance enabled precise localization of the mouse cervix and uterine body, reducing the need for excessive cervical dilation. Procedures were performed under direct visualization, allowing accurate control of instrument position and force, thus avoiding additional uterine injury, such as perforation or hemorrhage. Consequently, this approach offers greater safety, less trauma, and faster operation times.

However, transvaginal mechanical injury guided by ultrasound requires operator proficiency with both ultrasound and surgical techniques, and differences in complication rates may be more pronounced with operators have undergone standardized training and verification. Multicenter validation and larger sample sizes may help further assess and reduce such variability. Moreover, although few complications were observed in this study, this conclusion is limited by the small sample size and the short 14-day follow-up period, which may not capture delayed or infrequent adverse events. Additionally, this study used nonpregnant mice, whereas clinical IUA commonly occurs after repeated abortions and curettage following miscarriage [4]. Therefore, some discrepancies may exist between the present model and pathophysiology observed patient. IUA development is also influenced by multiple factors, such as hormonal fluctuations, postpartum physiological changes, and intrauterine infections. while this study focused on primarily on the mechanical injury component. These clinical contributors were not incorporated into the present model, which focuses primarily on the mechanical injury component of adhesion formation. As these clinical contributors were not incorporated, the model does not fully recapitulate the complex environment of human IUA. Nonetheless, although the method effectively induces adhesion formation, further optimization—such as incorporating postpartum or hormonally primed conditions or introducing infection-related inflammation—may help establish a more clinically relevant model.

Regarding the control group, the animals underwent laparotomy without intrauterine injury. However, abdominal surgery itself can induce a certain degree of inflammation or even adhesions, potentially affecting baseline comparisons and limiting the ability to distinguish effects caused exclusively by the intrauterine injury. A control group without abdominal surgery may therefore provide a more accurate baseline for future studies and better isolate the specific contributions of intrauterine mechanical injury to adhesion formation.

## 5 Conclusion

We successfully established an intrauterine adhesion (IUA) model in mice using transvaginal mechanical injury guided by abdominal ultrasound. This method demonstrated high clinical relevance, accurately replicating key pathological features of IUA, including endometrial thinning, glandular loss, inflammation, and fibrosis. Compared with traditional laparotomy or absolute ethanol models, the ultrasound-guided approach resulted in fewer early and late experimental complications, preserved uterine integrity, and allowed precise procedural control under direct visualization.

Overall, this model provides a reliable and safe platform for studying IUA pathogenesis and evaluating potential preventive and therapeutic strategies. Its adoption may improve the translational relevance of preclinical studies in reproductive medicine.
